# Reduction of the ATPase inhibitory factor 1 (IF_1_) leads to visual impairment in vertebrates

**DOI:** 10.1038/s41419-018-0578-x

**Published:** 2018-06-04

**Authors:** Rebeca Martín-Jiménez, Danilo Faccenda, Emma Allen, Holly Beatrice Reichel, Laura Arcos, Caterina Ferraina, Daniela Strobbe, Claire Russell, Michelangelo Campanella

**Affiliations:** 1 Department of Comparative Biomedical Sciences, Royal Veterinary College, NW1 0TU London, United Kingdom; 2 Department of Biology, University of Rome Tor Vergata, 00144 Rome, Italy; 3 IRCCS- Regina Elena, National Cancer Institute, 00133 Rome, Italy; 4 University College London Consortium for Mitochondrial Research, University College London, WC1 6BT London, United Kingdom

## Abstract

In vertebrates, mitochondria are tightly preserved energy producing organelles, which sustain nervous system development and function. The understanding of proteins that regulate their homoeostasis in complex animals is therefore critical and doing so via means of systemic analysis pivotal to inform pathophysiological conditions associated with mitochondrial deficiency. With the goal to decipher the role of the ATPase inhibitory factor 1 (IF_1_) in brain development, we employed the zebrafish as elected model reporting that the *Atpif1a*^−/−^ zebrafish mutant, pinotage (*pnt*^*tq209*^), which lacks one of the two IF_1_ paralogous, exhibits visual impairment alongside increased apoptotic bodies and neuroinflammation in both brain and retina. This associates with increased processing of the dynamin-like GTPase optic atrophy 1 (OPA1), whose ablation is a direct cause of inherited optic atrophy. Defects in vision associated with the processing of OPA1 are specular in *Atpif1*^−/−^ mice thus confirming a regulatory axis, which interlinks IF_1_ and OPA1 in the definition of mitochondrial fitness and specialised brain functions. This study unveils a functional relay between IF_1_ and OPA1 in central nervous system besides representing an example of how the zebrafish model could be harnessed to infer the activity of mitochondrial proteins during development.

## Introduction

The zebrafish (*Danio rerio*) represents a powerful model system for studying vertebrate development and the pathogenesis of human diseases (as reviewed in ref.^1^). Recently, it has also been employed as a tool for mitochondrial genetic and pharmacological studies^2,3^. Mitochondrial defects are commonly observed in a wide spectrum of human pathologies, such as cancer, diabetes and neurodegeneration. As in humans and mammals, the phenotypes observed in zebrafish include neuronal and synapse loss^4^, alterations in brain activity^5^, aberrant motor and sensory responses^5^, blood and vascular disorders^6^. Mitochondrial disorders can be caused by either defective mitochondrial bioenergetics or intracellular transport, which can result in abnormal subcellular localization of the organelle, increased production of reactive oxygen species (ROS) or impaired OXPHOS activity^7^. The zebrafish mitochondrial respiratory chain complexes have a high degree of identity with the human counterparts and their inhibition causes severe developmental defects, ranging from morphological and physiological abnormalities to embryonic arrest^2^. Brain development is tightly regulated by the metabolic state of mitochondria whose mass may change during neuronal differentiation. Indeed, while a decrease in mitochondrial activity has been observed as human embryonic stem cells (hESCs) differentiate into neural stem cells (NSC)^8^, mitochondrial biogenesis increases during the maturation of NSCs into motor neurons^9^. Trafficking of mitochondria is also crucially involved in the formation of axons, dendrites and synaptic connections^10^.

The F_1_F_o_-ATPsynthase plays an important part in all this. Physiologically, its activity regulates cellular ATP provision^11^, mitochondrial ultrastructure^12^, Ca^2+^ handling and cell death^13^. Defects in the function and assembly of the F_1_F_o_-ATPsynthase, are observed in postnatal and age-related neurometabolic disorders (i.e. Leigh syndrome, maternally inherited Leigh syndrome, Leber hereditary optic neuropathy and neuropathy, ataxia, and retinitis pigmentosa^14^), all of which characterized by early-onset and motor and sensory neurological symptoms, such movement disorders and visual impairments^15^. The activity of this enzyme is indeed involved in driving synapse formation and sustaining physiological brain activity, and its impairment associates with a decline in both neuronal performance and plasticity^16^.

Aging processes are not exception as loss of mitochondrial inner membrane organization following disassembly of F_1_F_o_-ATPsynthase complexes is also described^17^.

The ATPase inhibitory factor 1 (IF_1_), which is the most characterized regulator of the F_1_F_o_-ATPsynthase^18,19^, binds to the enzyme inhibiting its hydrolytic activity thereby protecting cells from ATP depletion during de-energized conditions^18^. A series of evidences suggests that, in cancer cells, the deregulated, enhanced binding of IF_1_ to the F_1_F_o_-ATPsynthase activates oncogenic regulatory mechanisms^20–22^. IF_1_ though is mainly known for its protective role from hypoxic/ischaemic damage in tissues with high-energy demand, such as heart and brain^23^. However, its ubiquitous expression and high degree of sequence conservation underline a wider role, which has been recently associated with the regulation of stem cell differentiation^24^, hepatic cholesterol uptake^25^, haem synthesis^6^, cell proliferation and programmed demise^26,27^.

IF_1_ expression is sustained in neurons^28^, which highly rely on oxidative metabolism, which is per se indicative of a core role in energy provision, which is not reported in astrocytes^29^. The upregulation of IF_1_ expression is notably observed during brain preconditioning, involving adaptation of both mitochondrial metabolism^30^ and quality control^23,31–33^. Equally, deregulation of IF_1_ activity could harm these processes, leading to the onset of pathological conditions. We therefore set to investigate this using the *Atpif1a*^−/−^ zebrafish mutant *pinotage* (*pnt*^*tq209*^), which is indeed lacking of the *a* gene type of the *Atpif1* paralogues.

The zebrafish genome contains two copies of the *Atpif1 gene*, *Atpif1a* and *Aatpif1b*^6^, a duplication that is likely to originate in teleosts^34^ and so encode for two proteins with partially redundant functions^6^. We have recently used the zebrafish mutant *pnt*^*tq209*^ to study the involvement of IF_1_ in haem synthesis reporting reduced rate of ferrochelatase-dependent iron incorporation into protoporphyrin^6^ leading to decreased haem content and erythrocyte volume^6^. Whether IF_1_ expression affects other systemic functions and organ physiology during development is nonetheless unknown. Here we report that alterations in IF_1_ expression lead to visual impairments in both zebrafish larvae and mice providing evidences in complex model systems of an underlying interplay between IF_1_ and OPA1 in the definition of mitochondrial homoestasis exploited during development and reflected systemically.

## Results

### Reduced IF_1_ levels lead to increased apoptosis and neuroinflammation in the brain and retina

The analysis of the effect of reduced IF_1_ levels on zebrafish neurodevelopment started with an examination of nervous system cell survival and proliferation during embryogenesis in the *pnt*^*tq209*^ mutant. Indeed, an imbalance between proliferation and apoptosis leads to defective clonal expansion of progeny cells, abnormal organ growth and functional impairments. For the purpose, we first determined the number of apoptotic bodies in both brain and retina of *pnt*^*tq209*^ mutants and normal siblings (Sbs) at 72 h post fertilisation (hpf).

**Fig. 1:**
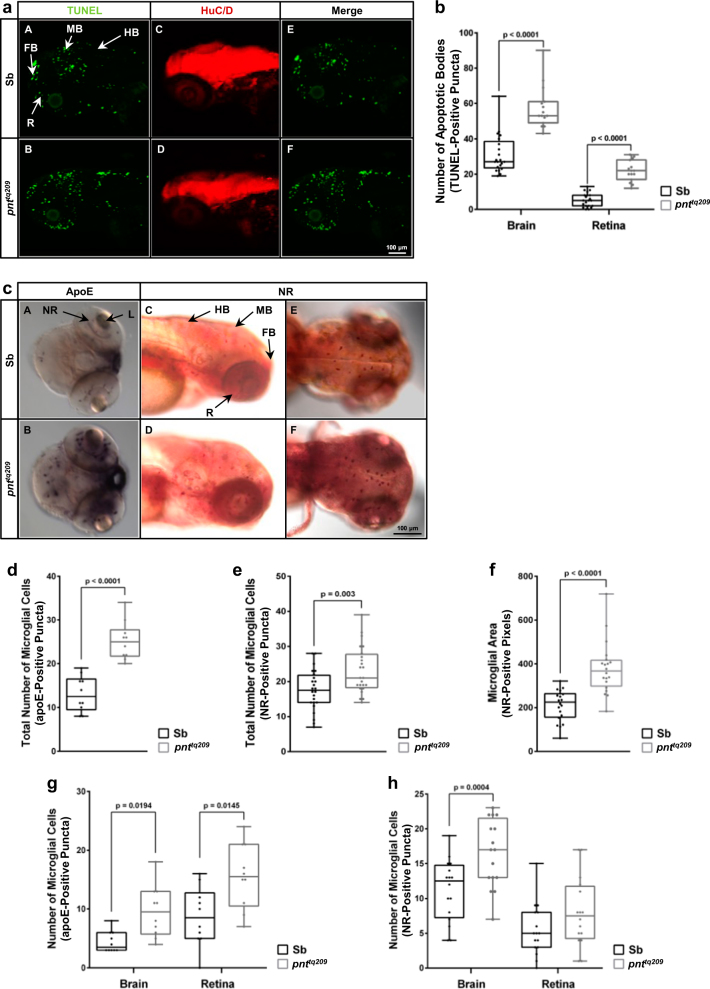
Apoptosis and microglial activation are increased in the CNS of *pnt*^*tq209*^ larvae. **a** TUNEL assay (**A**, **B**) and HuC/D (**C**, **D**) immunostaining of PTU-treated normal Sb and *pnt*^*tq209*^ mutant zebrafish at 72 hpf to detect apoptotic cells and differentiated neurons, respectively. The merge between the two fluorescent signals is shown in **E** and **F**. **b** Quantification of apoptotic bodies, detected with the TUNEL assay, in Sb and *pnt*^*tq209*^ zebrafish, showing a significant increase in the total number of apoptotic bodies in the brain and retina of mutant larvae (number of apoptotic bodies (TUNEL-positive puncta) in the brain, Sb: 31.10 ± 2.35, *pnt*^*tq209*^: 57.53 ± 3.12; in the retina, Sb: 5.33 ± 0.85, *pnt*^*tq209*^: 21.80 ± 1.52; results are reported as mean ± S.E.M. (*n* = 15–20)). **c** *apoE* in situ hybridization (**A**, **B**) and NR staining (**C**–**F**) were carried out in PTU-treated, 72 hpf zebrafish to visualise microglia. **d**, **e** Quantification of *apoE*-positive (**d**) and NR-positive (**e**) cells in the whole CNS. A significant increase in the number of microglial cells characterizes *pnt*^*tq209*^ zebrafish ((number of *apoE*-positive puncta, Sb: 13.10 ± 1.28, *pnt*^*tq209*^: 25.20 ± 1.38; number of NR-positive puncta, Sb: 17.50 ± 1.18, *pnt*^*tq209*^: 23.13 ± 1.35; results are reported as mean ± S.E.M. (*apoE*: *n* = 10; NR: *n* = 24)). **f** Analysis of the size of NR puncta. *pnt*^*tq209*^ zebrafish showed a significant increase in the size of microglia when compared to Sb (microglial area (NR-positive pixels), Sb: 210.45 ± 15.65, *pnt*^*tq209*^: 379.35 ± 26.73; results are reported as mean ± S.E.M. (*n* = 15)). **g**, **h** Evaluation of the number of cells stained with *apoE* (**g**) and NR (**h**) in the brain and retina, separately. The results demonstrate that, in *pnt*^*tq209*^ larvae, the increase in microglial cell population is also extended to the retina ((number of *apoE*-positive puncta in the brain, Sb: 4.40 ± 0.56, *pnt*^*tq209*^: 9.60 ± 1.38; in the retina, Sb: 8.70 ± 1.58, *pnt*^*tq209*^: 15.60 ± 1.73; number of NR-positive puncta in the brain, Sb: 11.19 ± 1.10, *pnt*^*tq209*^: 16.50 ± 1.21; in the retina, Sb: 5.44 ± 0.93, *pnt*^*tq209*^: 7.94 ± 1.23; results are reported as mean ± S.E.M. (*apoE*: *n* = 10; NR: *n* = 15)). FB forebrain, HB hindbrain, MB midbrain, R retina

Larvae were subjected to TUNEL staining prior to HuC/D immunostaining to simultaneously visualize apoptotic bodies and neuronal cells, respectively (Fig. 1a). The analysis, conducted with a confocal microscope, revealed an increase in the number of apoptotic bodies in both brain and retina of *pnt*^*tq209*^ mutants (Fig. 1b), even though no obvious differences in the pattern of the neuronal marker HuC/D were observed between mutants and Sb (Fig. 1a).

Considering the increased rate of programmed cell death observed in the *pnt*^*tq209*^ mutants, we hypothesized that this could lead to a neuroinflammatory response. Therefore, we monitored the level of microglial activation in both mutants and Sb. For the purpose, we employed in situ hybridisation (ISH) of *apolipoprotein E* (*apoE*) mRNA and neutral red (NR) vital dye staining^35,36^ to visualize microglia in the *pnt*^*tq209*^ zebrafish brain (Fig. 1c). Interestingly, we found an increase in both *apoE*-positive (Fig. 1d, g) and NR-positive (Fig. 1e, h) cells in the brain and retina of *pnt*^*tq209*^ larvae. Microglial cells did also appear significantly larger (Fig. 1f), thus implying an activated state^37^. Taken together, these data suggest the induction of an inflammatory response in *pnt*^*tq209*^ zebrafish as ablation of *Atpif1a* expression may lead to cell loss and a neuroinflammatory phenotype in zebrafish.

### IF_1_ loss causes visual impairment in *pnt*^*tq209*^ zebrafish and *Atpif1*^−/−^ mice

**Fig. 2:**
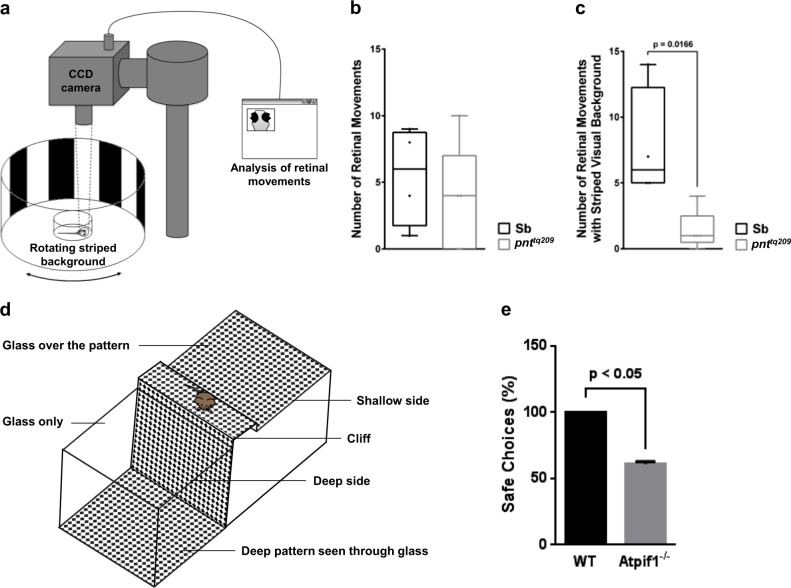
Vision is impaired in zebrafish and mice lacking Atpif_1_. **a** Schematic representation of the device used to measure the OKR of zebrafish larvae. **b**, **c** OKR in Sb and *pnt*^*tq209*^ zebrafish at 72 hpf. While normal and mutant larvae have a comparable number of retinal movements in normal conditions (**b**), the latter exhibit a significantly reduced response to visual stimulation, which was achieved by a rotating striped disc (**c**) (number of retinal movements in normal conditions, Sb: 5.5 ± 1.85, *pnt*^*tq209*^: 3.5 ± 1.58; with striped visual background, Sb: 7.75 ± 2.14, *pnt*^*tq209*^: 1.40 ± 0.40; results are reported as mean ± S.E.M. (*n* = 5)). **d** Model of the visual cliff apparatus. **e** Visual cliff test in WT and *Atpif1*^−/−^ mice. The quantification of safe choices (avoidance of the drop) indicates that visual acuity is mildly compromised in mutant mice (percentage safe choices, WT: 100.00 ± 0.00, *Atpif1*^−/−^: 61.00 ± 1.73); results are presented as mean ± S.E.M. (*n* = 5))

Excessive, uncontrolled microglial activation is a major cause of inflammation-mediated neurodegeneration^38^. As higher levels of apoptotic bodies were detected in both brain and retina of *pnt*^*tq209*^ mutants, and chronic neuroinflammation is frequently associated with retinal neurodegeneration^39^, we decided to further explore the phenotypic outcome of *Atpif1a* loss by assessing the visual function in *pnt*^*tq209*^ larvae. For this purpose, the optokinetic response (OKR) of *pnt*^*tq209*^ and normal Sb zebrafish larvae was measured. The OKR is the ocular movement induced by changes in the visual surround, and is commonly used to obtain an accurate quantitative readout of visual ability in zebrafish larvae^40^. At 72 hpf, the larval OKR was assessed by counting the number of eye movements under normal conditions or during constant visual stimulation. Each record lasted 3 min, and visual stimulation was achieved by moving black and white stripes around the test chamber (Fig. 2a). Remarkably, the analysis revealed a different response to the moving striped pattern between *pnt*^*tq209*^ mutants and normal Sb. Even though *pnt*^*tq209*^ larvae did not show any noticeable alteration under normal conditions, they were significantly less sensitive to constant light changes in the surrounding environment (Fig. 2b, c). This indicates that *Atpif1*a deficiency is associated with mild vision impairment, which affects the responsiveness of *pnt*^*tq209*^ larvae to visual stimuli.

To corroborate this finding, we monitored the visual acuity in 4-month-old *Atpif1*^−/−^ mice. This was investigated by using the visual cliff apparatus, monitoring the propensity by mice to step towards the platform side (safe choice) and avoid the drop (Fig. 2d). Notably, the loss of *Atpif1* correlated with alterations in the spatial perception and a substantial reduction in the number of positive choices was observed in *Atpif1*^−/−^ mice (Fig. 2e), a response that also characterizes strains affected by retinal degeneration^31^.

The data collected so far confirm that *Atpif1* contributes to correct visual function in vertebrates. The protein can therefore be ascribed among the mitochondrial factors that play a role in the development and maintenance of the neural retina, among which the dynamin-like protein optic atrophy 1 (OPA1) is the most known^37^. Mutations in the OPA1 gene that impair the expression or activity of the protein are associated with hereditary optic neuropathies related with mitochondrial dysfunction, such as autosomal-dominant optic atrophy (ADOA)^41,42^. Interestingly, although ubiquitously expressed throughout the body, during embryonic development IF_1_ is significantly upregulated in the retina of both zebrafish^6^ and mouse^43,44^. Furthermore, IF_1_ shares a similar chronological expression profile during development with OPA1^45^ and recently we described a functional relay between IF_1_ and OPA1, which shields the latter from the processing mediated by the Metalloendopeptidase OMA1^46^.

**Fig. 3:**
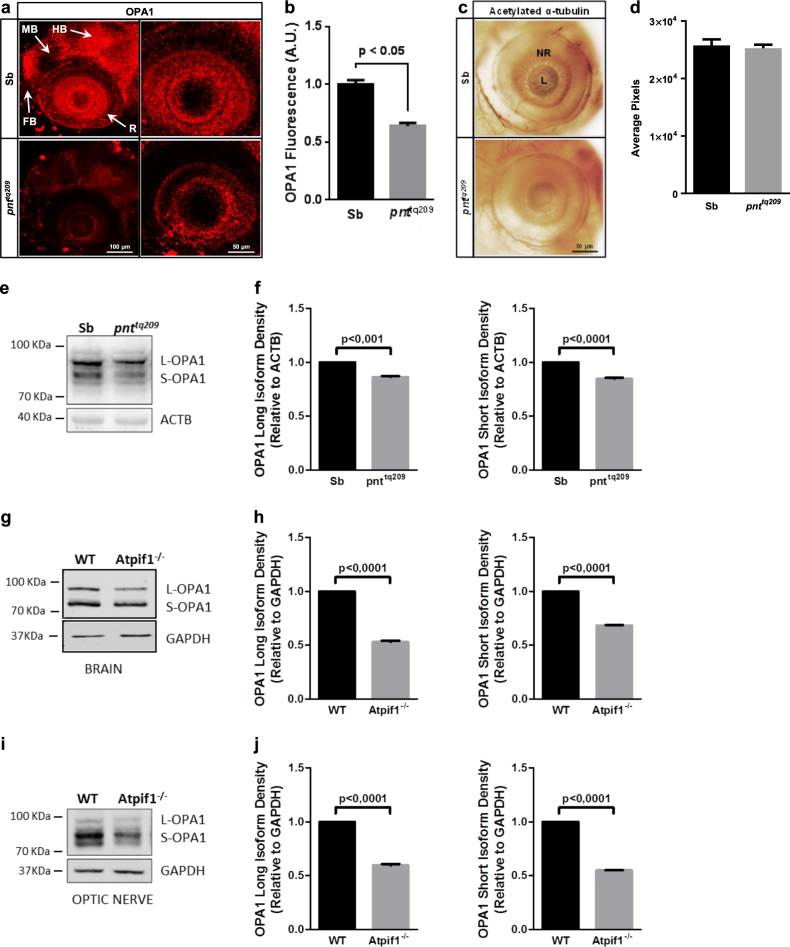
Atpif_1_ deficiency causes a decline in the retinal expression of OPA1. **a**, **b** Fluorescent IHC of whole-mount 72 hpf zebrafish, stained with anti-OPA1 antibody. Representative images (**a**) and quantification of OPA1 fluorescence intensity (**b**) are reported, showing a reduction in the OPA1 expression levels in the brain and retina of *pnt*^*tq209*^ larvae (OPA1 fluorescence (A.U.), Sb: 1.00 ± 0.01, *pnt*^*tq209*^: 0.70 ± 0.09; results are presented as mean ± S.E.M. (*n* = 3)). **c**, **d** Analysis of eye morphology in Sb and *pnt*^*tq209*^ zebrafish obtained through anti-acetylated α-tubulin IHC of whole-mount 72 hpf zebrafish. No major morphological differences were observed between normal and mutant larvae, as shown in the prototypical images (**c**) and relative quantification of eye size (**d**) (eye size (pixels), Sb: 2.58 ± 0.10, *pnt*^*tq209*^: 2.53 ± 0.06; results are presented as mean ± S.E.M. (*n* = 10)). **e**, **f** Quantification of OPA1 levels via western blotting analysis in 72 hpf wild type and *pnt*^*tq209*^ larvae. The representative membrane blotted for both OPA1 isoform (**e**) and the bar chart (**f**) show a significant decrease in the levels of the short isoform of the protein in *pnt*^*tq209*^ larvae. **g**–**j** (OPA1 band density relative to ACTB, OPA1 long isoform Sb: 1.00 ± 0.01, pnt^*tq209*^:0.97 ± 0.01; OPA1 short isoform Sb: 1.00 ± 0.01, pnt^tq209^:0.84 ± 0.01; results are presented as mean ± S.E.M. (*n* = 3), Quantitative western blot analysis of OPA1 levels in the brain (frontal cortex/hippocampus) (**j**, **k**) and isolated optic nerve (**l**, **m**) of WT and *Atpif1*^−/−^ mice. Representative blots (**j**, **l**) and quantitated band densities relative to GAPDH (**k**, **m**) are reported. Significantly lower levels of short and long OPA1 isoforms expression were found in the brain and, specifically, in the optic nerve of mutant mice (OPA1 band density relative to GAPDH, OPA1 long isoform WT: 1.00 ± 0.01, *Atpif1*^−/−^: 0.52 ± 0.01 in the frontal cortex/hippocampus; in the optic nerve, WT: 1.00 ± 0.05, *Atpif1*^−/−^: 0.59 ± 0.09; in the frontal cortex/hippocampus OPA1 short isoform WT: 1.00 ± 0.01, Atpif^1−/−^: 0.68 ± 0.01; in the optic nerve, WT: 1.00 ± 0.05, Atpif1^−/−^: 0.548 ± 0.01; results are presented as mean ± S.E.M. (*n* = 3). FB forebrain, HB hindbrain, MB midbrain, NR neural retina, L lens

In order to investigate whether the vision defects detected in zebrafish and mice lacking IF_1_ expression were related to OPA1 inactivation, we first monitored the levels of OPA1 in the brain and retina of *pnt*^*tq209*^ and normal Sb zebrafish at 72 hpf. The analysis was carried out by whole-mount immunofluorescence and revealed an extensive decrease in the expression profile of the protein in both brain (midbrain-hindbrain, in particular) and retina of *pnt*^*tq209*^ mutants when compared to Sb (Fig. 3a, b). Despite this, no prominent differences in the eye morphology (size and shape) were detected between *pnt*^*tq209*^ and wild-type (WT) larvae (Fig. 3c, d), implying that *Atpif1a* loss does not cause any major anatomical eye defects, and that mainly affects the neural part of the organ. To confirm this observation, the levels of both OPA1 isoforms were quantified via western blotting in the whole embryos (Fig. 3e, f). This analysis reports a decrease in the levels of the profusion protein, which prevalently affects the short isoforms. However, we should consider that the samples were obtained from the whole *pnt*^*tq209*^ larvae, entailing technical limitations in protein extraction by homogenization.

A tissue-specific analysis was instead obtained using extracts from WT and *Atpif1*^−/−^*mouse*. In these, OPA1 isoforms have been monitored in the brain (frontal cortex/hippocampus), optic nerve and liver homogenates (Fig. 3g-j, SFigure 1a,b). Even in this case, the expression of short and long OPA1 isoforms was downregulated in *Atpif1*^−/−^ mice (almost halved when compared to WT mice). This data suggest that, even though *Atpif1* deficiency causes a tissue-specific phenotypic alteration leading to visual impairment^6,47^, the physiological OPA1 processing seems to be retained in each tissue investiagated. Considering the close link between OPA1 loss-of-function and defects in optic nerve morphogenesis, which are a direct consequence of impaired mitochondrial function^48^, we further investigated the effect of IF_1_ loss on mitochondrial respiratory capacity. For the purpose, we evaluated the levels and assembly of mitochondrial respiratory chain complexes by two-dimensional blue native/SDS gel electrophoresis. Mitochondrial bioenergetic efficiency and adaptive capacity rely on the dynamic supramolecular organization of respiratory chain complexes in supercomplexes (SCs) or respirasomes. The analysis of the stoichiometric composition of mitochondrial respirasomes gives significant information on the organelle bioenergetic homoeostasis.

We attempted to measure the relative levels of SCs in extracts from WT and *Atpif1*^−/−^ murine liver and brain, as well as WT and IF_1_ knockdown human neuroblastoma SHSY-5Y cells. The analysis revealed that IF_1_ expression levels alters the respirasome assembly in both murine and human derived lines (S Figs. 1 and 2). Most notably, differences appear prominent in the layout of the complexes in which association between complexes I and III is far less in extracts in which the *Atpif1* gene is ablated or the protein product downregulated. We also note an overall reduction of the complex IV levels, which could per se reduce the respiratory efficiency, as well as favouring the accumulation of ROS and therefore trigger apoptosis.

All this is nonetheless indicative of an underlying mitochondrial phenotype associated with *Atpif1* loss and aberrant processing of OPA1, which could lead to the defects in visual capacity observed in both zebrafish and mice.

### Analysis of locomotor ability in *pnt*^*tq209*^ zebrafish

**Fig. 4:**
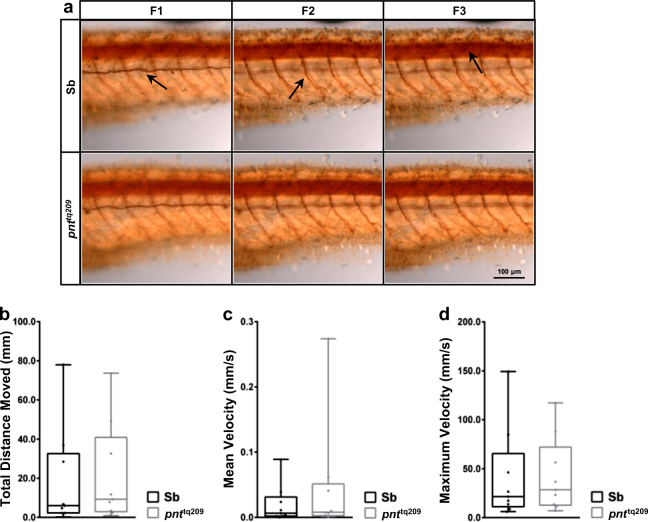
*pnt*^*tq209*^ zebrafish show normal development of the spinal cord and locomotor ability. **a** Anti-acetylated α-tubulin IHC of whole-mount 72-hpf zebrafish. No marked differences in axonal growth and morphology were observed between Sb and *pnt*^*tq209*^ larvae. Images were acquired at three different focal planes to visualize the lateral line (F1) and the ventral and dorsal motor axons (F2 and F3, respectively). **b**–**d** Locomotor activity assay in 72-hpf zebrafish. Total distance moved (**b**), mean velocity (**c**) and maximum velocity (**d**) were measured (average total distance moved (mm), Sb: 18.39 ± 8.60, *pnt*^*tq209*^: 21.22 ± 8.46; average mean velocity (mm/s), Sb: 0.02 ± 0.01, *pnt*^*tq209*^: 0.05 ± 0.03; average maximum velocity (mm/s), Sb: 41.57 ± 15.74, *pnt*^*tq209*^: 42.47 ± 12.62; results are reported as mean ± S.E.M. (*n* = 9)). No variances were observed between Sb and *pnt*^*tq209*^ larvae

Based on the results described above, we examined whether locomotion could also be affected by IF_1_ deficiency due to axonal alterations in the caudal region of the larvae. The number and morphology of spinal cord motor axons were monitored via immunostaining of acetylated α-tubulin (Fig. 4a). The absence of noticeable differences between *pnt*^*tq209*^ and normal Sb zebrafish larvae suggests that IF_1_ loss does not significantly affect the neurogenesis of spinal cord motor neurons. Nonetheless, we carried out a quantitative assay of larvae locomotion to assess whether motor neuron function was compromised. The total distance moved by mutant and WT larvae, together with their mean and maximum velocity, were analysed (Fig. 4b-d). The assay demonstrated no significant motility alteration of *pnt*^*tq209*^ mutants, indicating that zebrafish lacking *Atpif1*a does not exhibit typical signs of motor neuron degeneration.

## Discussion

This study examined the role of IF_1_ in the development of the zebrafish nervous system during embryogenesis. Our interest stemmed from the notion that IF_1_ expression, which is maintained at high levels in adult neurons^30,49^, undergoes timed regulation during differentiation and maturation of stem cells^24,50^, suggesting a possible role for the protein in vertebrate development. This hypothesis appears to be confirmed by the defects in erythroblast differentiation associated with *Atpif1a* loss in zebrafish, which cause a severe form of sideroblastic anaemia^14^. Nonetheless, little is still known about the effect of pathological alterations in IF_1_ expression during embryogenesis. The zebrafish is an extremely valuable model system for studying vertebrate biology, also enabling developmental genetic and functional studies^51^. Moreover, considering the high level of conservation of genetic sequences, molecular processes and organ systems between zebrafish and other vertebrates, including humans, zebrafish is emerging as preferred model for studying developmental pathology^52^. In this work, the use of the *pnt*^*tq209*^ zebrafish mutant allowed the identification of alterations in the neural system, attributable to loss of *Atpif1a* expression. Specifically, we detected cell loss (i), induction of a pro-inflammatory environment (ii) and vision impairment (iii).

During brain maturation, apoptosis plays a key role and any defect in the process, such as irregular, premature and/or excessive apoptosis, can lead to neurodegeneration^53^. We recently reported that the level of IF_1_ expression defines the cellular response to apoptotic stress preventing cellular demise^22,26^. Here we found that the *pnt*^*tq209*^ mutant zebrafish is characterized by a significant increase in the number of apoptotic bodies in both brain and retina, even though no significant alterations were observed in the pattern of differentiated neurons between mutant and WT larvae (Fig. 1).

The abnormal levels of apoptosis that characterize *pnt*^*tq209*^ zebrafish seem to have an impact on the activity of microglial cells, which represent the resident macrophages of the CNS and are involved in the maintenance of cell homeostasis by clearing dead and dysfunctional neurons. *pnt*^*tq209*^ zebrafish are characterized by higher microglial activity (Fig. 1), which can be a direct consequence of the increased programmed demise both in the brain and retina of *pnt*^*tq209*^ mutants. Though microglia play a neuroprotective role^36^, their excessive activity is frequently observed in damaged brain tissues and neurodegenerative disorders^38,54^.

By using specific microglial markers, we identified an increase in both number and size of microglial cells in *pnt*^*tq209*^ mutant zebrafish confirming that phagocytic activity of microglia is enhanced in the absence of *Atpif1a* and causes neuroinflammatory damage. Notably, activated microglial cells are localized at high levels in the retina of the larvae (Fig. 1).

The concomitant rise in both cell death and microglia-induced neuroinflammation detected in the retina of *pnt*^*tq209*^ larvae suggests that organ function may also be compromised. By testing the OKR of *pnt*^*tq209*^ larvae (Fig. 2), we gained evidence that IF_1_ deficiency associates with visual impairment noting a significant reduction in retinal movements triggered by changes in the surrounding environment (Fig. 2). A decline in visual acuity was also observed in *Atipif1*^*−/−*^ mice, corroborating that IF_1_ may have a conserved role in the development of vertebrate vision (Fig. 2).

The retinal photoreceptor cells, which comprise two different types of neurons, cones and rods, both specialized in phototransduction, are metabolically active cells characterized by high-mitochondrial content^55,56^. Tissues with high-energy demand, such as the neural retina, are frequently affected by the presence of mutations in genes that encode for mitochondrial proteins^57^. The impairments in visual capacity caused by IF_1_ deficiency in zebrafish and mouse may be therefore related to retinal mitochondrial alterations. Indeed, IF_1_ controls different aspects of mitochondrial homeostasis^6,20,26,58^ and mitochondrial defects are commonly associated with eye disorders^59^.

A mitochondrial protein that is highly involved in vertebrate embryonic development is the dynamin-like GTPase OPA1^60,61^. OPA1, which exists as long, inner membrane-bound, and short, soluble, isoforms, forms high-molecular weight complexes that localize at the cristae junctions and control both the fusion of the inner membranes of two merging mitochondria and the shaping of mitochondrial cristae^62^. OPA1 also plays a prominent part in cristae remodelling and cytochrome *c* release during apoptosis^63^. Recently, we discovered that IF_1_ has similar activity that relies on stabilization of the mitochondrial ultrastructure^22^, a function that exerted in coordination with OPA1^47^. Alterations in mitochondrial dynamics and metabolism have been previously proposed as a possible mechanism of the OPA1-type ADOA pathogenesis^64^. Moreover, OPA1 deficiency causes developmental defects in zebrafish, including abnormal cardiac function and blood circulation^60^. With our study, we provide further proof of shared pathological mechanisms between IF_1_ and OPA1. Both are highly expressed in the developing vertebrate brain and retina^43–45,65^ and we learnt that IF_1_ interplays with OPA1 in the control of mitochondrial adaptation to programmed cell death^46^. Here we show that impairment of this relay, which is core to mitochondrial structure and functions affects the visual capacity in both models of analysis leaving unaffected other parametres such as the locomotion at least at the state of analysis here adoboted^66,67^.

Beyond the pathophysiological outcome, which we linked to OPA1, the modifications in the mitochondrial complexes and SCs assembly recorded via the in-gel analysis corroborate how IF_1_ is intimately linked with mitochondrial OXPHOS homeostasis (S Figs. 1 and 2) beyond the preservation of ATP pools during reversion of the ATPsynthase^28^.

**Fig. 5:**
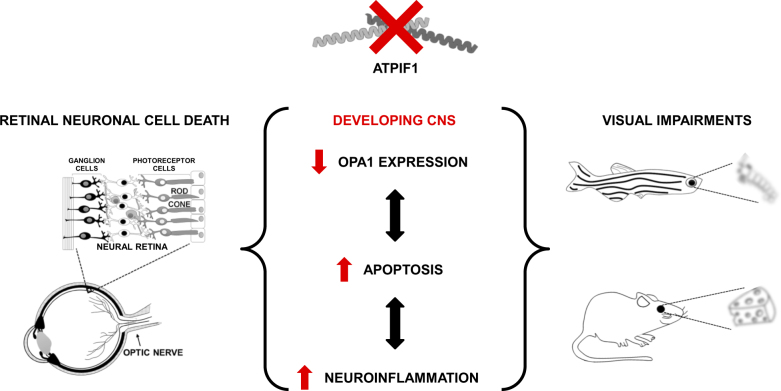
Involvement of IF_1_ in vertebrate eye development. The loss of IF_1_ activity causes abnormally high apoptosis and microglial activation in the CNS and retina during zebrafish embryogenesis, an event which suggests increased levels of neuroinflammation. IF_1_ deficiency is also associated with a marked decline in the brain and retinal levels of OPA1, the expression of which is fundamental for guaranteeing the correct development of the vertebrate eye. As a consequence, IF_1_ KO zebrafish and mouse models are both characterized by mild visual impairment that does not interfere with the normal animal functions, and which is possibly caused by apoptosis- and neuroinflammation-mediated neuro-retinal damage

Though in zebrafish the loss of IF_1_ induces detectable alterations solely in their visual capacity, we cannot exclude endogenous compensatory mechanisms mediated by the *Atpif1a*-related paralogue, *Atpif1b*, which might reduce or mask the effects of the IF_1_ loss in other tissues. However, the lethal phenotype of the double *Atpif1a/b* KO zebrafish mutant^6^ prevented further in-depth analysis. The merit of this work does lie in its capacity to reveal how IF_1_ takes part the developmental of central nervous system widening the relevance of this mitochondrial protein (see working model depicted in Fig. 5) beyond the pathological processes to which it is prevalently associated^26,32,68^.

## Materials and methods

### Animal models

Zebrafish were housed in a multi-rack aquarium system at the Royal Veterinary College and kept on a constant 14/10 h light/dark cycle at 27–29 °C^69^. The *pnt*^*tq209*^ mutant zebrafish line^6^ was obtained from the Tübingen Stock Center. Zebrafish embryos, obtained by natural spawning, were examined and manually dechorionated under a Nikon SMZ1500 microscope (Nikon, Kingston upon Thames, UK). To prevent pigmentation, embryos were raised in water containing 0.003% 1-phenyl-2-thiourea (PTU), starting at 24 hpf. At 72 hpf, larvae were categorized as either normal Sb or *pnt*^*tq209*^ mutants depending on their phenotype^6^. All zebrafish experiments were locally ethically approved by the Royal Veterinary College, and nationally approved by the UK Home Office under the Animal (Scientific Procedures) Act 1986.

C57 BL/6 (WT) and C57/BL6J (*Atpif1*^−/−^) mice^47^ were used in accordance with national and European (86/609/EEC) guidelines.

### TUNEL and anti-HuC/D immunofluorescence double-labelling assay

DeadEnd™ Fluorometric TUNEL System (Promega, G3250) was used for the specific detection and quantitation of apoptotic cells in PTU-treated and dechorionated 72 hpf zebrafish. The catalytic incorporation of fluorescein-12-dUTP at 3′-OH DNA ends was developed following the principle of the TUNEL (TdT-mediated dUTP Nick-End Labelling) assay and the manufacturer recommendations. Briefly, 72 hpf zebrafish were fixed in 4% paraformaldehyde (PFA) in PBS (overnight incubation at 4 °C). After fixation, larvae were washed three times in PBS + 0.1% Triton™ X-100 (PBT) and then subjected to proteinase K (Sigma-Aldrich, P2308) digestion (15 ng/ml for 1 h in PBT). Subsequently, larvae were fixed for 20 min in 4% PFA in PBS, blocked in equilibration buffer for 30 min, and finally incubated for 1 h at 37 °C in TdT reaction mix (equilibration buffer + nucleotide mix and rTdT enzyme in a 5:1 ratio). Unincorporated fluorescein-12-dUTP was removed through three washes in PBT. After TUNEL assay was performed, larvae were incubated for 1 h at room temperature in blocking solution (10% normal goat serum plus 1% dimethyl sulphoxide in PBT), and then overnight at 4 °C with a mouse anti-HuC/D antibody (1:1000; Molecular Probes, A21271). Following several washes in PBT, the larvae were incubated overnight at 4 °C with a goat anti-mouse Alexa Fluor^®^568-conjugated antibody (1:400; Invitrogen, A11004). Fluorescently-labelled larvae were imaged using a laser-scanning confocal microscope (Leica^TM^ SP5). All images were acquired without changing the microscope settings and processed in the same way. The number of apoptotic bodies in the brain and retina was quantified using ImageJ software (NIH).

### Immunohistochemistry (IHC) of whole-mount zebrafish larvae

IHC was performed in whole-mount PTU-treated and dechorionated 72 hpf zebrafish as previously described^70^. Larvae immunostained with anti-acetylated α-tubulin (1:1000; Sigma-Aldrich, T7451)^70^ were imaged with a Zeiss Axiovert inverted microscope.

For anti-OPA1 fluorescent IHC, PTU-treated and dechorionated 72 hpf zebrafish were fixed in 4% PFA in PBS and treated with proteinase K (15 ng/ml for 1 h in PBS + 0.1% Triton™ X-100). After leaving the larvae in blocking solution for 1 h, they were incubated overnight at 4 °C with a mouse anti-OPA1 antibody (1:500; BD Biosciences, 612607). Following a wash in PBT for several hours, larvae were then incubated overnight at 4 °C with a goat anti-mouse Alexa Fluor^®^568-conjugated antibody (1:400; Invitrogen, A11004). Larvae were cleared in 70% glycerol in PBS and imaged with a laser-scanning confocal microscope (Leica^TM^ SP5). OPA1 fluorescent intensity was quantified using ImageJ software.

### NR assay

Microglial cells were detected in living dechorionated zebrafish larvae by staining with NR, a vital dye that is accumulated in the lysosomes through endocytosis^35^. PTU-treated and dechorionated 56 hpf zebrafish were incubated in the dark overnight (to reach the larval stage) at 28–30 °C in 2.5 μg/mL NR solution. After two rinses with fish water, larvae were fixed in 2% low melting-point agarose in fish water. Live imaging was carried out using a Zeiss Axiovert inverted microscope. The number and size of NR-positive cells in the brain and retina were quantified using ImageJ software.

### Whole-mount ISH of *apoE* mRNA

ISH was carried out on PTU-treated and dechorionated 72 hpf zebrafish using an *apoE* riboprobe (a generous gift from Dr. Francesca Peri, European Molecular Biology Laboratory, Heidelberg, Germany), as previously described^36^. The number of *apoE*-positive cells in the brain and retina was quantified using ImageJ software (NIH).

### Western blot analysis of mouse brain extracts

Four-month-old mice were killed by cervical dislocation and, prior to dissection, sterilized with ethanol. The skull was opened to release the brain, which was immediately stored on ice. The frontal cortex, hippocampus and optic nerve were then isolated and lysed in radioimmunoprecipitation assay buffer (50 Mm Tris, 150 mM NaCl, 1 mM EDTA, 5 mM MgCl2, 1% Triton™ X-100, 0.25% sodium deoxycholate, 0.1% SDS, pH 7.4) supplemented with protease/phosphatase inhibitors (Roche Diagnostics, 04693132001). The lysates obtained from the frontal cortex and hippocampus were then combined. The lysates were sonicated and centrifuged at 17,000×*g* at 4 °C for 20 min, after which the supernatants were collected and stored at −80 °C.

The protein concentration was estimated using a BCA protein assay reagent (Thermo Scientific). Equal amounts of protein (20 μg) were resolved in 8% polyacrylamide gel and transferred to nitrocellulose membrane. The membrane was blocked in 3% non-fat dry milk in TBST (50 mM Tris, 150 mM NaCl, 0.05% Tween 20 (Sigma-Aldrich), pH 7.5) for 1 h and then incubated with the appropriate diluted primary antibody at 4 °C overnight: mouse anti-OPA1 (1:1000; BD Biosciences, 612607), mouse anti-GAPDH (1:10000; Abcam, ab 8245). After three washes in TBST, the membrane was incubated with goat anti-mouse HRP-conjugated antibody for 1 h at room temperature. Amersham ECL Prime Western Blotting Detection Reagent kit (GE Healthcare Life Sciences, RPN2232) was used to develop the membrane. Immunoreactive bands were analysed with ImageJ software (NIH).

### In-gel analysis of mitochondrial respirasomes

The method was adapted from Nijtmans et al.^71^, Wittig et al.^72^ and Calvaruso et al.^73^. Mice were killed by cervical dislocation. Then, brain and liver tissues were collected on ice and homogenized using a glass-Teflon potter homogenizer in mitochondrial isolation buffer (440 mM sucrose, 20 mM Mops, 1 mM EDTA) with 0.2 mM phenylmethylsulfonyl fluoride. Protein extracts were separated by Blue Native Bis-Tris PAGE 4–16% (NativePage Thermo Fisher scientific).

Homogenates were centrifuged at 20,000×*g* for 15 min at 4 °C. After that the pellet was homogenized in a solution composed of 1 M aminocaproic acid and 50 mM Bis-Tris-HCL pH 7.0. Digitonin 4 mg/mL was added to the homogenate and incubated for 20 min on ice. After centrifugation at 100,000×*g* for 15 min, the supernatant was collected and combined with Serva blue G 5% in 1 M aminocaproic acid.

The Coomassie Blue Staining was obtained by immersing the gels in staining solution 0.1% Serva G in 40% methanol for 30 min at room temperature. Then the staining solution was removed and the gels were incubated with destaining solution 40% methanol and 10% acetic acid for 30 min twice. At this point the gels were scanned.

### Locomotor activity assay

Dechorionated 72 hpf zebrafish were individually placed into the wells of a 96-well plate. A DMx21AF04 digital camera was used with Noldus 2010 Media Recorder software to record 20 min videos with a frame rate of 30 frames/s and 640 × 480 resolution. The videos were processed using EthoVision XT8 software (Noldus) to track the movements of each zebrafish larva. Quantitive data was produced using three movement parameters: total distance moved (mm), mean velocity (mm/s) and maximum velocity (mm/s).

### OKR measurements

Three minute, 30 fps digital recordings of 72 hpf zebrafish were taken using a DMx21AF04 digital camera with Noldus 2010 Media Recorder software. Each animal was recorded with and without a moving striped visual background made from black and white cards, where each stripe was ~2 cm thick. In recordings using the striped visual background, card was rotated around the 96-well plate in small back and forth movements in quick succession. One retinal movement includes one or both retinae.

### Visual cliff test

Visual acuity was tested in C57 BL/6 (WT) and C57/BL6J *Atpif1*^−/−^ mice using the visual cliff test according to Carlezon and colleagues^74^. The apparatus comprised a 1 m height checkered pattern platform covered with a clear piece of acrylic glass, extended by 50 cm from the edge of the platform, and a checkered pattern sheet positioned below the extending glass. A raised dais was placed between the platform and the extending glass, creating the illusion of a cliff. Each mouse was placed on the raised dais and allowed to step off to either the shallow side (safe choice) or the deep side (unsafe choice). Each mouse was tested in ten trials, and choices were manually recorded as safe if the mouse stepped towards the platform and unsafe if the mouse stepped towards the extending acrylic glass.

### Statistics

Statistical analyses were performed using GraphPad Prism 6 software. Two groups were compared using the unpaired *t*-test. Comparison of three or more groups was performed by one-way ANOVA. A *p*-value <0.05 was considered significant.

## Electronic supplementary material

Supplementary Figures

Supplementary Legend
