# Supplementary figures and images for: Reduction of the ATPase inhibitory factor 1 (IF1) leads to visual impairment in vertebrates
Source: Cell Death Dis. 2018 Jun 4;9(6):669. doi: 10.1038/s41419-018-0578-x (PMC5986772; doi:10.1038/s41419-018-0578-x)

# Supplementary Figure 1

**a**

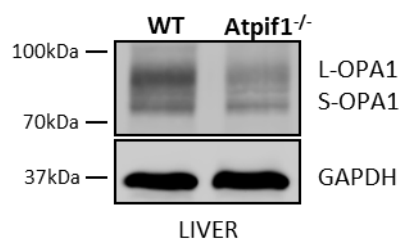

**b**

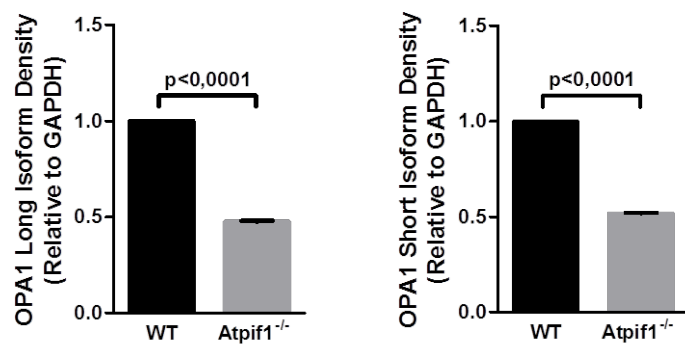

**c**

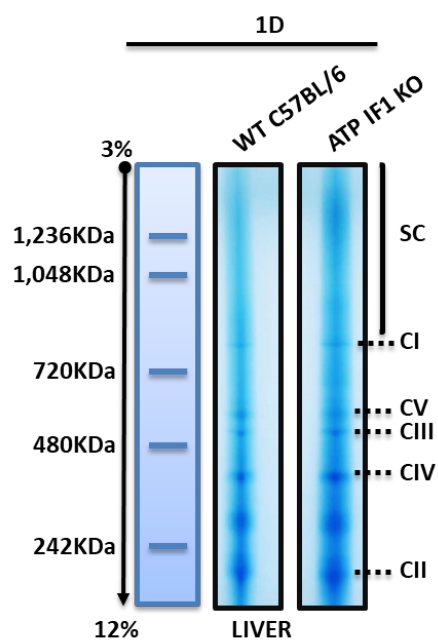

**d**

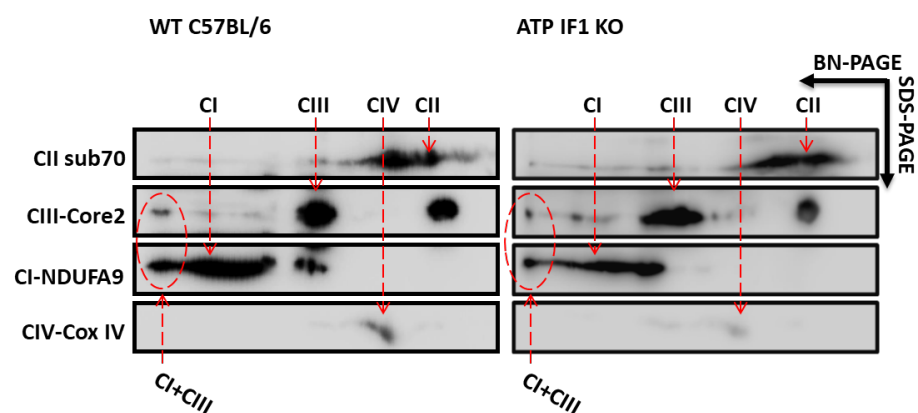

**e**

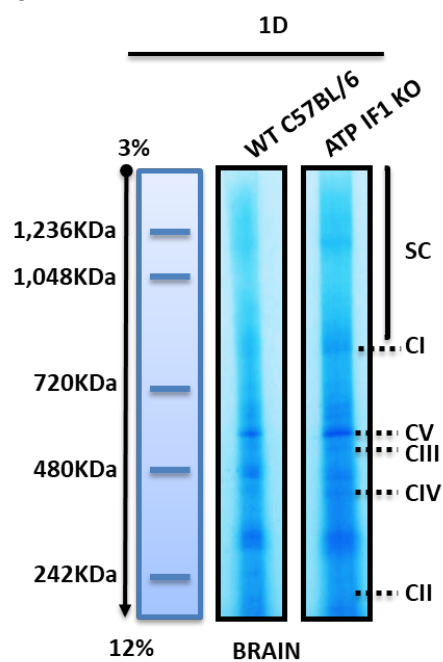

**f**

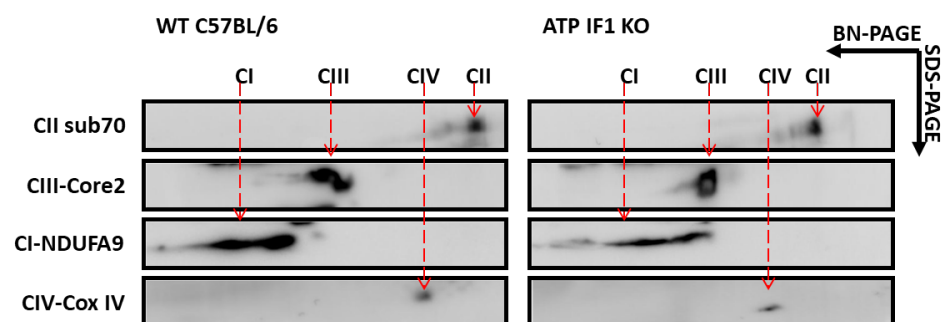

Supplementary Figure 2

a

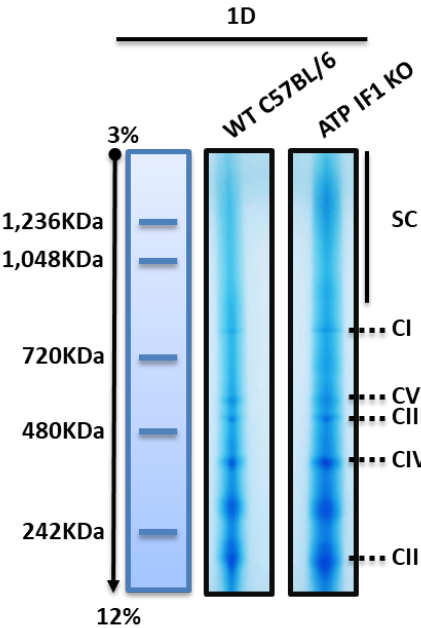

b

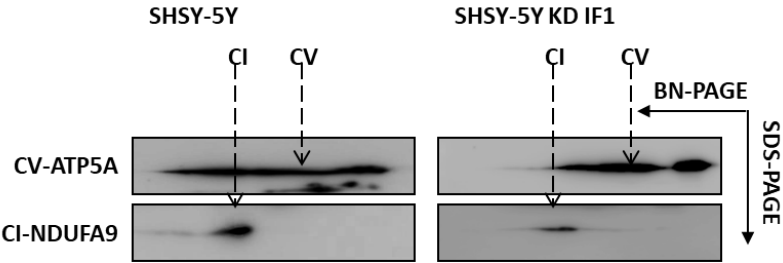

Supplement: Supplementary file 1 — Supplementary Figures [file 41419_2018_578_MOESM1_ESM.pdf]
