# Supplementary material for: Reduction of the ATPase inhibitory factor 1 (IF1) leads to visual impairment in vertebrates
Source: Cell Death Dis. 2018 Jun 4;9(6):669. doi: 10.1038/s41419-018-0578-x (PMC5986772; doi:10.1038/s41419-018-0578-x)
Supplement: Supplementary file 2 — Supplementary Legend [file 41419_2018_578_MOESM2_ESM.docx]

**Supplemetary Figure 1. Assesment of OPA-1, complexes and supercomplexes in brain and liver extract of wild type and *Atpif1^-/-^* mice**

Quantitative Western blot analysis of OPA1 levels in the liver of WT and *Atpif1^-/-^* mice (a). Representative blots and quantitated band densities relative to GAPDH (b) are reported. Significantly lower levels of short and long OPA1 isoforms expression were found in the liver (OPA1 band density relative to GAPDH, OPA1 long isoform WT: 1.00 ± 0.01, *Atpif1*^-/-^: 0.47 ± 0.01; OPA1 short isoform WT: 1.00 ± 0.05, *Atpif1*^-/-^: 0.51 ± 0.01; results are presented as mean ± S.E.M. (n = 3). Solubilized liver (c) and brain (e) mitochondrial complexes (I-V) were separated according to their indicated masses on a linear 3-12% gradient gel for BN-PAGE. (d-f) Representative blot of OXPHOS system complexes resolved by second dimension denaturing SDS-PAGE. Spots were detected with antibodies against the indicated subunits.

**Supplementary Figure 2. Analysis of complexes lay-out in cell lines extracts.**

Blue native GEL from human SHSY-5Y and SHSY-5Y stably downregulated for IF_1_. Solubilized mitochondrial complexes (I-V) were separated according to their indicated masses on a linear 3-12% gradient gel for BN-PAGE.
